# Combined antiviral therapy as effective and feasible option in allogenic hematopoietic stem cell transplantation during SARS-COV-2 infection: a case report

**DOI:** 10.3389/fonc.2024.1290614

**Published:** 2024-02-13

**Authors:** Serena Vita, Alessandra D’Abramo, Andrea Coppola, Chiara Farroni, Anna Paola Iori, Francesca Faraglia, Alessandro Sette, Alba Grifoni, Cecilia Lindestam Arlehamn, Michele Bibas, Delia Goletti, Emanuele Nicastri

**Affiliations:** ^1^ Clinical Department, National Institute for Infectious Diseases ‘Lazzaro Spallanzani’ Istituti di Ricovero e Cura a Carattere Scientifico (IRCCS), Rome, Italy; ^2^ Policlinico Umberto I, Sapienza University of Rome, Rome, Italy; ^3^ Center for Infectious Disease and Vaccine Research, La Jolla Institute for Immunology (LJI), La Jolla, CA, United States

**Keywords:** immunocompromised, SARS-CoV-2 infection, HSCT, antivirals, prolonged infection, dual antiviral therapy, antigen-specific response

## Abstract

Here we describe the case of a 51 years old Italian woman with acute lymphoblastic leukemia who underwent to hematopoietic stem cell transplantation (HSCT) during SARS-COV-2 infection. She presented a prolonged COVID-19 successfully treated with dual anti SARS-COV-2 antiviral plus monoclonal antibody therapy.

## 1 Introduction

Allogeneic hematopoietic stem cell transplantation (HSCT) is considered as a definitive treatment option for several hematologic disorders. HSCT recipients are at high risk of severe COVID-19 with a mortality rate ranging from 22% to 45% (1). Therefore, the European Bone Marrow Transplantation guidelines recommend HSCT deferral until patients are asymptomatic with two negative molecular nasopharyngeal swab (m-NPS) for SARS-CoV-2.

Here we describe the case of an Italian woman with acute lymphoblastic leukemia who underwent HSCT during SARS-COV-2 infection who was successfully treated by dual anti-SARS-CoV-2 antiviral plus monoclonal antibody (MoAb) therapy.

## 2 Case report

On January 28 2023, a 51 year old female patient with SARS-CoV-2 infection and a history of acute lymphoblastic leukemia, B common, was admitted to the National Institute for Infectious Diseases Lazzaro Spallanzani-IRCCS in Rome, Italy. She was fully vaccinated for SARS-CoV-2 with BNT162b2 (3 doses, last dose on April 2022), with no previous SARS-CoV-2 natural infection.

On May 2022, she received chemotherapy according to “GIMEMA” (Gruppo Italiano Malattie Ematologiche dell’Adulto) LAL1913 protocol. Due to the persistence of minimal residual disease (MRD), she underwent blinatumomab therapy to attempt MRD negativity before starting HSCT procedure.

On October 25, the m-NPS for SARS-CoV-2 resulted negative and then the day after was admitted to Hematology Department of Policlinico Umberto I, “Sapienza” University of Rome, to receive HSCT. On November 8 a further m-NPS resulted negative. On November 9^th^ she started pre-transplant conditioning regime with total body irradiation (12 Gy) and Fludarabine. Cyclosporine, mycophenolate mofetil and post-transplant cyclophosphamide were used for GVHD prophylaxis. On November 15, she developed a pauci-symptomatic, with a hacking cough, and the m-NPS confirmed a SARS-CoV-2 BA.5.5 infection with 20 Cycle Threshold (CT). The pt was treated with a 10-day full therapy with intravenous (iv) remdesevir for 10-days (200 mg the first day followed by 100 mg for 9 days) and the thorax computer tomography scan (CT-scan) was negative. On November 18^th^, she underwent to HSCT from a mismatched unrelated volunteer donor. Donor was fully vaccinated with 3 doses of SARS-COV-2 vaccine (BioNTech/Pfizer), the last one in march 2022. Engraftment in polymorphonuclear cells was observed on day 19 after transplant. On December 16^th^, she was discharged fully asymptomatic, with a still positive m-NPS for SARS-CoV-2. On December 28^th^, flu-like symptoms were reported, the m-NPS was still positive with 21 CT, and a CT-scan evidence of bilateral interstitial pneumonia as for SARS-COV-2 infection. Moreover a diffuse inflammatory thickening of the maxillary sinuses: voriconazole therapy was started (200 mg BID) in the suspicious of fungal infection. Few days later, acute GVHD-grade II with skin lesions, was diagnosed: steroid treatment (1 mg/kg prednisolone) was daily given for 14 days and then tapered. The patient over the next 30 days was clinically stable only with serotinous fever.

**Figure 1:**
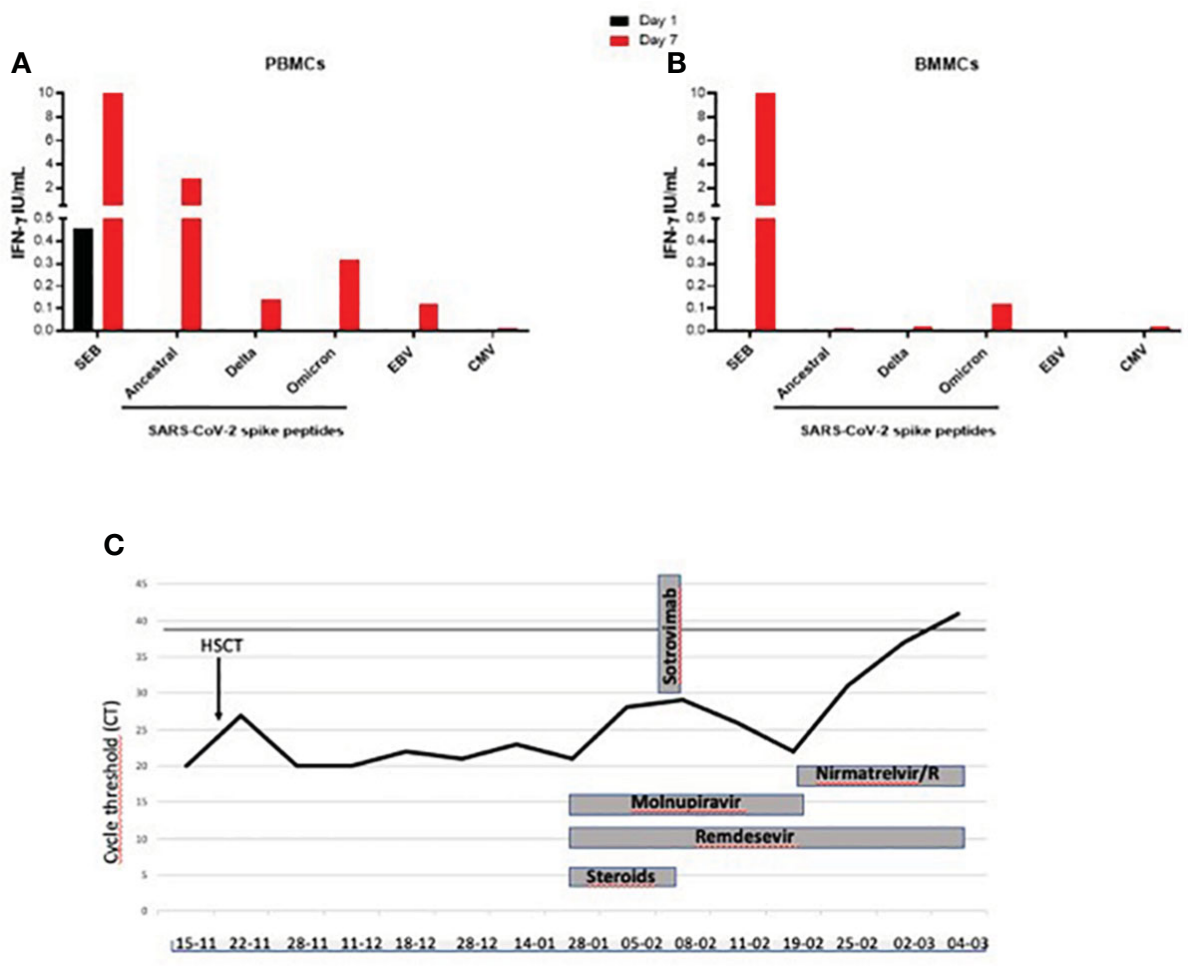
**(A–C)**. T cell-specific and unspecific response evaluated by the detection of IFN-γ in PBMC and BMMC and evaluation of the timeline of virological replication during anti-SARS-CoV-2 treatments in a COVID-19 patient receiving an allogenic hematopoietic stem cell transplantation. Black line in **(C)** indicates the m-NPS negativity. IFN-γ, Interferon-γ; SEB: staphylococcal enterotoxin B; Spike: SARS-CoV-2 CD4 pool of peptides; DELTA: B.1.617.2 SARS-CoV-2 variant, OMICRON: B.1.1.529 SARS-CoV-2 variant; EBV: Epstein-Barr virus; CMV: cytomegalovirus.

At the admission in our ward on January 28^th^ 2023 the m-NPS showed a 21 CT value. Hypogammaglobulinemia (Immunoglobulin IgG 226 mg/dl) and absent CD19 cells were reported. She was under oral treatment with cyclosporine (50 mg twice daily), letermovir (240 mg daily), voriconazole (200 mg twice daily), valaciclovir (500 mg daily), cotrimoxazole (160/800 mg twice weekly), prednisolone (25 mg daily). She started a dual anti-SARS-CoV-2 therapy with oral molnupiravir and iv remdesivir (200 mg the first day followed by 100 mg for 35 days), followed by iv sotrovimab, a monoclonal antibody (MoAb) against SARS-CoV-2 glycoprotein. On February 15, a bone marrow aspirate was performed for HSCT follow-up monitoring, with paired peripheral and medullar blood collection. A coordinated B and T cell immunity is needed to control SARS-CoV-2 replication (2). Therefore, we evaluated if, although in the absence of SARS-CoV-2 antibody production, a T-cell specific response was detectable (3, 4). In PBMC we confirmed the absence of CD19^+^ B cells by flow cytometry (data not shown). Unfortunately, we could not evaluate it in BMMC due to the small amount of medullar blood. The T cell response was assessed at the time of sampling on February 15, by *in vitro* stimulation with an unspecific stimulus as SEB, and with specific stimuli using peptides of SARS-CoV-2 and also with Epstein Barr Virus (EBV) and Cytomegalovirus (CMV) peptides to evaluate a recall response to non-Corona Viruses, as described in the methods section. The response was evaluated in PBMCs and BMMCs at day 1 and 7 post *in vitro* stimulation (5). In the PBMCs and BMMCs at day 1 (black bar) we detect a T cell response only to SEB. At day 7 in PBMCs, after a long-term *in vitro* stimulation (red bar), we found a rescued SARS-CoV-2-specific response against spike from the ancestral strain and from delta and omicron variants (**Figure 1A**); also EBV-specific response was recovered. Differently, in BMMCs at day 7 we found only a weak and selective positive response to the spike from the omicron variant (**Figure 1B**).

T cell response to unspecific (SEB) and specific stimuli (SARS-CoV-2, EBV, CMV) was evaluated by the IFN-γ detection in (a) PBMCs and (b) BMMCs at day 1 (black square) and day 7 (red square) post *in vitro* stimulation. For the long-term stimulation, IL-2 was added at day 3 post-*in vitro* stimulation and culture supernatants were harvested at day 7. The IFN-γ values shown are subtracted from the value recorded in the unstimulated control. (c) Virologic test results from m-NPS and COVID-19 treatments are reported. Treatments included monoclonal infusions (sotrovimab), remdesivir (200 mg first day followed by 100 mg die intravenous), molnupiravir (800 mg bid per os) and nirmatrelvir/ritonavir (300 mg/100 mg die bid per os). Steroid doses below 0.1 mg/kg prednisone equivalents are indicated.

On February 21^th^, the m-NPS cycles were 22 and molnupiravir was stopped and nirmatrelvir/ritonavir, an antiviral with a different target, was added to remdesevir. Voriconazole was replaced by isovuconazole (200 mg daily) to avoid drug-drug interaction with ritonavir (**Figure 1C**), and cyclosporine plasma level was monitored every 48 hours. On March 4, the m-NPS was negative and she was discharged. At 4-month follow-up period, no SARS-CoV-2 relapse, or breakthrough infection were reported.

## 3 Patient and method

Peripheral blood mononuclear cells (PBMCs) and Bone Marrow mononuclear cells (BMMCs) were collected during SARS-CoV-2 infection. Cells *were in vitro* overnight (day 1) or long-term (for 7 days) stimulated with Staphylococcal Enterotoxin B (SEB, 200 ng/mL), SARS-CoV-2 spike peptides (ancestral strain; delta and omicron variant of Concern (VoC) (5, 6) and with Epstein Barr (EBV) and Cytomegalovirus (CMV) peptides (1µg/mL each). Briefly, the peptide megapool design was carried out on the Wuhan-Hu-1 reference isolated (GenBank ID: MN908947) and consists of 253 overlapping 15-mers by 10 spanning the entire spike protein (CD4-S; n = 253); VR13 DELTA B.1.617.2 (1µg/mL); VR14 OMICRON B.1.1.529 (1µg/mL)]. For the long-term stimulation (day 7), IL-2 (5UI/mL, from R&D, Minneapolis, MN 55413) was added at day 3 post*-in vitro* stimulation and culture supernatants were harvested at day 7.

The specific IFN-γ response was evaluated by ELISA according to manufacturer’s instructions (www.quantiFERON.com). The IFN-γ values shown are subtracted from the values recorded in the unstimulated control.

B cell phenotype was evaluated in whole blood with the B-cell Tubes (BD Biosciences, San Jose, California (CA), USA) and the frequency of CD45^+^ and total B cells (CD19^+^) were assessed.

Real-time reverse transcription polymerase chain reaction (RT-PCR) was performed according to the laboratory workflow using Alinity m SARS-CoV-2 Assay (Abbott, Chicago, Illinois, United States) targeting RdRp and N genes. SARS-CoV-2 variants identification was conducted by Sanger sequencing of the Spike coding gene.

## 4 Discussion

Autologous and allogenic HSCT is a potentially curative therapy in several high-risk hematologic malignancies and HSCT has been performed even during COVID-19 pandemic, although several groups and societies advised that non-urgent bone marrow transplants could be delayed on single case-by-case assessment.

In the context of COVID-19 pandemic, HSCT recipients are at increased risk of mortality which is largely dependent on age, comorbidities, active hematologic disease, timing from transplant and severity of the infection (1, 7). Moreover, patients undergoing HSCT or receiving cellular therapies may shed viable SARS-CoV-2 for months (4, 8).

We describe the case of a female patient with acute lymphoblastic leukemia who performed HSCT after 3 days from a pauci-symptomatic SARS-CoV-2 infection. Despite SARS-CoV-2 infection, HSCT was considered an undeferrable therapeutic option during the aplasia phase induced by conditioning regimen.

Forty days later the patient developed an interstitial pneumonia and one month later, she was hospitalized for SARS-COV-2 persistence (21 CT at m-NPS). CT-values between 17 and 32 represent an amount of viable virus that is usually considered to be replicative-competent and infectious (9).

No specific guidelines for the treatment of prolonged COVID-19 in IC setting have been proposed, no randomized clinical trials have been published and all treatment approach are based on anecdotical clinical cases (4, 8, 10).

In the first part of COVID-19 pandemic when MoAbs were highly active against SARS-CoV-2 VoC, a common approach was the combined antiviral and MoAb therapy (8, 11) or hyper-immune COVID-19 convalescent plasma (CCP) (12). Later, MoAb neutralization activity decreased against the newly VOC and new therapeutic strategies were proposed. A double antiviral regime based on extended course of nirmatrelvir/ritonavir and remdesevir, eventually associated with CCP has been found effective in IC patients (4, 13, 14).

To date three direct-acting antivirals are approved for COVID-19 therapy in humans: remdesevir, molnupiravir, nirmatrelvir/ritonavir. These antivirals showed *in vitro* additive effect of nirmatrelvir/ritonavir and remdesivir or molnupiravir (15).

In IC setting, antiviral treatment choices are often influenced by drug-drug interactions. Our patient was initially on dual therapy with remdesevir and molnupiravir dual therapy, both drugs with few drug interactions. We used this combination since was already used with benefit in some anecdotal cases (16). Nevertheless, considering the viral persistence at high viral load in the m-NPS, molnupiravir was replaced by nirmatrelvir/ritonavir. To prevent potentially drug-drug interaction, the cyclosporine plasma level was monitored and voriconazole was replaced by isovuconazole. The immune results obtained indicate the presence of a memory T cell-specific response against SARS-CoV-2 ancestral and variant strains (17–20). SARS-CoV-2 infection persistence in this patient may be explained by the lack of B cells, since this can negatively affect virus neutralization and virus-specific CD8^+^ T cells expansion (19–21). Likely, the antiviral and monoclonal therapies combined with a residual presence of T cell-SARS-CoV-2-specific response were responsible for the good outcome of the patient.

## 5 Conclusions

In conclusion, the 35 days double antiviral regimen course, combined with both SARS-CoV-2 MoAb passive infusion and vaccine induced residual host-T cell specific response were all determinant factors of the favorable clinical outcome. Although SARS-CoV-2 infection during HSCT represents one of the worst clinical scenarios, this single case experience shows that a prolonged combined antiviral therapy can be an effective and feasible option to obtain SARS-CoV-2 viral clearance even in heavily IC COVID patients.

## Data availability statement

The original contributions presented in the study are included in the article/supplementary material. Further inquiries can be directed to the corresponding author.

## Ethics statement

The EC approval was not required, since it was a retrospective study. The studies were conducted in accordance with the local legislation and institutional requirements. The human samples used in this study were acquired from a by- product of routine care or industry. Written informed consent to participate in this study was not required from the participants or the participants’ legal guardians/next of kin in accordance with the national legislation and the institutional requirements. Written informed consent was obtained from the individual(s) for the publication of any potentially identifiable images or data included in this article.

## Author contributions

SV: Conceptualization, Investigation, Writing – original draft, Writing – review & editing. AD: Investigation, Writing – original draft. AC: Investigation, Writing – review & editing. CF: Investigation, Writing – review & editing. AI: Investigation, Writing – review & editing. FF: Investigation, Writing – review & editing. AS: Supervision, Writing – review & editing. AG: Validation, Writing – review & editing. CA: Validation, Writing – review & editing. MB: Writing – review & editing. DG: Funding acquisition, Supervision, Writing – review & editing. EN: Funding acquisition, Supervision, Validation, Writing – review & editing.
